# Supplementary material for: Socioeconomic inequalities in reach, compliance and effectiveness of lifestyle interventions among workers: protocol for an individual participant data meta-analysis and equity-specific reanalysis
Source: BMJ Open. 2019 Feb 13;9(2):e025463. doi: 10.1136/bmjopen-2018-025463 (PMC6398771; doi:10.1136/bmjopen-2018-025463)
Supplement: Supplementary data [file bmjopen-2018-025463supp001.pdf]

Supplementary file A. Search strategies for lifestyle interventions among workers

2018 February 19th

Search strategy in Embase (read from bottom-up).

No	Query
**#5**	#1 AND #2 AND #3 AND #4
**#4**	'Netherlands'/exp OR (Netherlands OR dutch):ab,ti,kw,ca,ta,cy,ad
**#3**	'workplace'/exp OR Employee/de OR employer/de OR worker/de OR (worker OR workplace* OR worksite* OR employee* OR employer* OR (work* NEAR/3 population*) OR 'at work'):ab,ti,kw
**#2**	'intervention study'/exp OR 'program evaluation'/exp OR 'education program'/exp OR 'smoking cessation program'/exp OR 'health education'/exp OR (intervention* OR program* OR (health NEAR/3 (education* OR promot*))):ab,ti,kw
**#1**	'lifestyle'/exp OR 'lifestyle modification'/exp OR 'sedentary lifestyle'/exp OR 'physical activity'/exp OR 'sitting'/exp OR exercise/exp OR sport/exp OR 'health behavior'/de OR 'alcohol consumption'/de OR 'drinking behavior'/exp OR 'smoking cessation'/exp OR obesity/exp OR 'body mass'/exp OR 'body weight'/de OR 'body weight change'/exp OR 'body weight control'/exp OR 'body weight gain'/exp OR 'body weight loss'/exp OR 'waist circumference'/de OR 'waist hip ratio'/de OR 'skinfold thickness'/de OR 'body fat'/de OR 'body composition'/de OR 'body distribution'/de OR 'body fat distribution'/de OR 'dietary intake'/exp OR 'healthy diet'/de OR 'unhealthy diet'/de OR 'feeding behavior'/exp OR (lifestyle OR 'life style' OR tobacco OR smoking OR smoker* OR cigarette* OR alcohol* OR addict* OR drinking OR sedentar* OR (physical* NEAR/3 (activ* OR inactiv*)) OR exercis* OR walking OR cycling OR jogging OR sitting OR running OR sport* OR obes* OR overweight* OR (body NEAR/3 (mass OR weight OR fat OR composition* OR distribut*)) OR (weight NEAR/3 (gain OR change OR loss OR reduc*)) OR overweight OR bmi OR (waist NEAR/3 (circumferen* OR hip)) OR (skinfold* NEAR/3 thick*) OR ((diet* OR eating OR feeding OR calor* OR fat) NEAR/3 (intake* OR behav* OR restrict* OR health* OR unhealth*)) OR 'fast food' OR fruit OR vegetable*):ab,ti,kw

Search strategy in Medline Ovid (read from bottom-up).

No	Query
**#5**	#1 AND #2 AND #3 AND #4
**#4**	Netherlands/ OR (Netherlands OR dutch).ab,ti,kw,jn,cp,in
**#3**	workplace/ OR (worker OR workplace* OR worksite* OR employee* OR employer* OR (work* ADJ3 population*) OR at work).ab,ti,kw.
**#2**	intervention studies/ OR exp program evaluation/ OR education/ OR education.xs. OR exp health education/ OR (intervention* OR program* OR (health ADJ3 (education* OR promot*))).ab,ti,kw.
**#1**	exp life style/ OR exp Motor Activity/ OR exp sports/ OR exp health behavior/ OR exp drinking behavior/ OR exp "Tobacco Use"/ OR exp obesity/ OR Body Mass Index/ OR exp Diet/ OR feeding behavior/ OR sports/ OR exp Running/ OR Bicycling/ OR body weight/ OR exp body weight changes/ OR Waist Circumference/ OR Waist-Hip Ratio/ OR skinfold thickness/ OR exp body composition/ OR Energy Intake/ OR healthy diet/ OR (lifestyle OR life style OR tobacco OR smoking OR smoker* OR cigarette* OR alcohol* OR addict* OR drinking OR sedentar* OR (physical* ADJ3 (activ* OR inactiv*)) OR exercis* OR walking OR cycling OR jogging OR sitting OR running OR sport* OR obes* OR overweight* OR (body ADJ3 (mass OR weight OR fat OR composition* OR distribut*)) OR (weight ADJ3 (gain OR change OR loss OR reduc*)) OR overweight OR bmi OR (waist ADJ3 (circumferen* OR hip)) OR (skinfold* ADJ3 thick*) OR ((diet* OR eating OR feeding OR calor* OR fat) ADJ3 (intake* OR behav* OR restrict* OR health* OR unhealth*)) OR fast food OR fruit OR vegetable*).ab,ti,kw.

Search strategy in Google scholar (read from bottom-up).

No	Query
**#1**	lifestyle|"life style"|smoking|alcohol|"physical activity"|obesity|overweight intervention|program|promotion worker|workplace|worksite|employee|employer Netherlands|dutch

Search strategy in Cochrane CENTRAL (read from bottom-up).

No	Query
**#5**	#1 AND #2 AND #3 AND #4
**#4**	Netherlands OR Dutch
**#3**	intervention* OR program* OR (health NEAR/3 (education* OR promot*)):ab,ti
**#2**	worker OR workplace* OR worksite* OR employee* OR employer* OR (work* NEAR/3 population*) OR 'at work'):ab,ti
**#1**	lifestyle OR 'life style' OR tobacco OR smoking OR smoker* OR cigarette* OR alcohol* OR addict* OR drinking OR sedentar* OR (physical* NEAR/3 (activ* OR inactiv*)) OR exercis* OR walking OR cycling OR jogging OR sitting OR running OR sport* OR obes* OR overweight* OR (body NEAR/3 (mass OR weight OR fat OR composition* OR distribut*)) OR (weight NEAR/3 (gain OR change OR loss OR reduc*)) OR overweight OR bmi OR (waist NEAR/3 (circumferen* OR hip)) OR (skinfold* NEAR/3 thick*) OR ((diet* OR eating OR feeding OR calor* OR fat) NEAR/3 (intake* OR behav* OR restrict* OR health* OR unhealth*)) OR 'fast food' OR fruit OR vegetable*):ab,ti

Search strategy in Web of science (read from bottom-up).

No	Query
**#5**	#1 AND #2 AND #3 AND #4
**#4**	Netherlands OR dutch
**#3**	intervention* OR program* OR (health NEAR/2 (education* OR promot*))
**#2**	worker OR workplace* OR worksite* OR employee* OR employer* OR (work* NEAR/2 population*) OR "at work"
**#1**	lifestyle OR "life style" OR tobacco OR smoking OR smoker* OR cigarette* OR alcohol* OR addict* OR drinking OR sedentar* OR (physical* NEAR/2 (activ* OR inactiv*)) OR exercis* OR walking OR cycling OR jogging OR sitting OR running OR sport* OR obes* OR overweight* OR (body NEAR/2 (mass OR weight OR fat OR composition* OR distribut*)) OR (weight NEAR/2 (gain OR change OR loss OR reduc*)) OR overweight OR bmi OR (waist NEAR/2 (circumferen* OR hip)) OR (skinfold* NEAR/2 thick*) OR ((diet* OR eating OR feeding OR calor* OR fat) NEAR/2 (intake* OR behav* OR restrict* OR health* OR unhealth*)) OR "fast food" OR fruit OR vegetable*

Search strategy in Web of science (read from bottom-up).

No	Query
**#5**	#1 AND #2 AND #3 AND #4
**#4**	Netherlands OR dutch
**#3**	intervention* OR program* OR (health NEAR/2 (education* OR promot*))
**#2**	worker OR workplace* OR worksite* OR employee* OR employer* OR (work* NEAR/2 population*) OR "at work"
**#1**	lifestyle OR "life style" OR tobacco OR smoking OR smoker* OR cigarette* OR alcohol* OR addict* OR drinking OR sedentar* OR (physical* NEAR/2 (activ* OR inactiv*)) OR exercis* OR walking OR cycling OR jogging OR sitting OR running OR sport* OR obes* OR overweight* OR (body NEAR/2 (mass OR weight OR fat OR composition* OR distribut*)) OR (weight NEAR/2 (gain OR change OR loss OR reduc*)) OR overweight OR bmi OR (waist NEAR/2 (circumferen* OR hip)) OR (skinfold* NEAR/2 thick*) OR ((diet* OR eating OR feeding OR calor* OR fat) NEAR/2 (intake* OR behav* OR restrict* OR health* OR unhealth*)) OR "fast food" OR fruit OR vegetable*
